# Nanolayer Growth on 3-Dimensional Micro-Objects by Pulsed Laser Deposition

**DOI:** 10.3390/nano11010035

**Published:** 2020-12-25

**Authors:** Nikolaos A. Vainos, Eleftherios Bagiokis, Vagelis Karoutsos, Jingshan Hou, Yufeng Liu, Jun Zou, Yongzheng Fang, Konstantina Papachristopoulou, Antonella Lorusso, Anna Paola Caricato, Alessio Perrone

**Affiliations:** 1 Department of Materials Science, University of Patras, 26504 Patras, Greece; ebagiokis@gmail.com (E.B.); vkar@upatras.gr (V.K.); k.papachristopoulou@upnet.gr (K.P.); 2 National Hellenic Research Foundation-TPCI, 48 Vass. Constantinou Ave., 11635 Athens, Greece; 3 School of Materials Science and Engineering, Shanghai Institute of Technology, Shanghai 201418, China; houjingshan@hotmail.com (J.H.); yfliu@mail.sitp.ac.cn (Y.L.); zoujun@sit.edu.cn (J.Z.); fyz1003@sina.com (Y.F.); 4 Dipartimento di Matematica e Fisica “E. De Giorgi”, Università del Salento, 73100 Lecce, Italy; antonella.lorusso@le.infn.it (A.L.); Annapaola.Caricato@le.infn.it (A.P.C.); alessio.perrone@le.infn.it (A.P.); 5 Istituto Nazionale di Fisica Nucleare, Sezione di Lecce, 73100 Lecce, Italy

**Keywords:** 3-dimensional coating, laser processing, pulsed laser deposition, nanocomposite, nanolayer, biopolymer, sensor, phosphor, light source

## Abstract

Pulsed laser deposition on 3-dimensional micro-objects of complex morphology is demonstrated by the paradigmatic growth of cellulose and polymer/Y_3_Al_5_O_12_:Ce phosphor composite nanolayers. Congruent materials transfer is a result of multicomponent ablation performed by relatively low fluence (<200 mJ cm^−2^) ArF excimer laser pulses (λ = 193 nm). Films grown on optical and engineering components, having a thickness from ~50 nm to more than ~300 nm, are durable, well adherent and maintain the structural and functional properties of the parent solids. The results verify the unique capabilities of deep-ultraviolet pulsed laser deposition of novel functional nanostructures on arbitrary surface morphologies and highlight its potential in future 3-dimensional nanotechnologies.

## 1. Introduction

Emerging 3-dimensional (3D) microdevices and micromachines for the life-sciences, sensing, and communication technologies [1] mandate the provision of advanced thin film functional coatings. Nanocomposite materials gain prime interest owing to their advanced, tunable and bespoke functionalities for novel mechanical, electrical, optical, and biological applications. The fabrication of such structures of complex surface morphology presents several challenges, among which, the coating of 3D microdevices is not a trivial task. In liquid-phase growth processes, for example, surface tension and capillary effects would affect the structural stability and destroy the object’s 3D topography and relief. Furthermore, a low-temperature, preferably a room-temperature, technique is needed to process heat-sensitive materials and maintain the functional properties. Finally, stricter requirements apply in the formation of synthetic hybrids and heterogeneous materials blends.

In view of the above, pulsed laser deposition (PLD) [2] is a most suitable candidate, having already demonstrated full compatibility with the above requirements. Operationally, the major advantage of laser ablation is the production of energetic multicomponent plasma, a source of electrons, ions, molecules, clusters, and larger particulates. These ejectiles, produced from solid or liquid targets, possibly composed of materials of different nature, travel in vacuum and upon incidence on a receiving substrate form a composite thin film with nanometric accuracy. The development of PLD in the past few decades has already demonstrated its flexibility to grow inorganic, organic, and hybrid layers on a broad range of solid substrates. Among relevant developments, we may highlight the very efficient metal photo-ejectors for high-energy particle accelerators [3], the epitaxial growth of waveguide lasers [4] and high-power laser devices [5], the oxide nanocomposites [6] and glass systems [7] for optoelectronics, to the recent advanced superconductor [8], oxide, boride, and nitride [9] materials. Furthermore, PLD of polymeric materials has been successfully demonstrated since the 1980s [10] and remarkable progress in the deposition of polymers and biomaterials has been made [11].

In the present work, we focus on the fabrication of 3D functional microdevices and report, for the first time to our knowledge, PLD on 3D micro-object surfaces of arbitrary stereometric form. To the best of our knowledge, PLD has been applied so far mainly using planar substrates, with only one exemption of silver and zinc-oxide antimicrobial coatings on micro-stereolithography-fabricated microneedle arrays [12]. In fact, major challenges are currently raised in the fabrication of biocompatible nanodevices, sensors, lab-on-chip and conformal light sources. Such issues we address here by demonstrating the paradigmatic growth of biopolymer and polymer-inorganic nanocomposite films on metallic engineering parts and polymer fibers, with an emphasis placed on biosciences and photonics.

In our first paradigm, we are concerned with the new class of biocompatible/biodegradable devices. We focus on cellulose, the most abundant polysaccharide, which is widely used in textile, paper, packaging, and medical industries, due to its remarkable structural stability, biocompatibility and biodegradability. Further to new commercial bio-phase fibers, films and membrane products, novel applications in tissue engineering [13], biosensing [14], photonics and electronics [15,16] are emerging. These advances have motivated our recent work on optical quality cellulose films [17] and prompted the present cellulose growth on model 3D objects mimicking biomedical components.

In the second paradigm, polymer–inorganic nanocomposites are considered. Metal–polymer PLD thin films have been demonstrated by sequential target ablation [18]. In a complementary alternative, we in-situ synthesized Ag-nanoparticle–polymer nanocomposite targets and directly transferred the material and grew plasmonic hybrids [19] for photonic sensing [20]. Extending these concepts to critical optoelectronic devices, we consider here the class of solid-state white-light LEDs (WLEDs) widely used in modern [21] and flexible lighting [22,23], industrial testing and sensing [24]. Among numerous types of phosphors, Y_3_Al_5_O_12_:Ce (YAG:Ce) garnet [25] is a principal representative. Promising trichromatic phosphors [26] and alternative light emitters [27] are developed and further promoted by current trends of visible-light communications [28]. Although rare-earth doped epitaxial Y_3_Al_5_O_12_ has been successfully grown by PLD [29] and recently phosphor YAG:Ce crystal has been reported [30,31], the high temperatures required for growth is clearly incompatible with our concept. Therefore, the application of polymer–phosphor composites is a unique solution. In contrast to concurrent multibeam PLD of phosphors and electro-optics [32,33] we demonstrate here, for the first time to our knowledge, congruent single-target growth of polymethylmethacrylate (PMMA)/YAG:Ce and polydimethylsiloxane (PDMS)/YAG:Ce nanocomposites on 3D micro-objects and observe photoluminescence of nanolayer-coated 3D engineering components and polymer fibers.

## 2. Materials and Methods

### 2.1. Experimental Configurations

**Figure 1:**
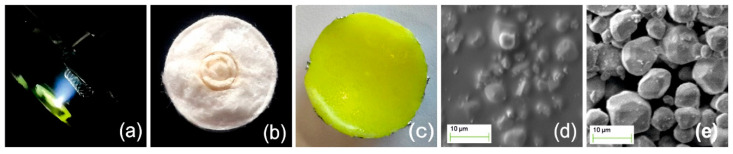
Photographic view of (**a**) PLD experiment of PMMA/phosphor coating a small metal spring. (**b**) PLD target of raw natural cotton. Two shallow ablation tracks are depicted. (**c**) PMMA/YAG:Ce PLD target. SEM images of: (**d**) target shown in (**c**), comprising phosphor microparticles embedded in PMMA matrix and (**e**) the as-prepared phosphor powder.

A high vacuum PLD reactor was used, equipped with oil-free/turbo pump system and quadrupole mass spectrometer to check the level and quality of the vacuum. The system incorporates optical ports and rotating sample and target holders. An ArF excimer laser PSX-501 Neweks (Neweks Ltd., Tallinn, Estonia) emitting deep ultraviolet laser pulses at λ = 193 nm, 5 ns pulse duration and maximum energy output 7 mJ per pulse, was used for ablation. Figure 1a presents a photographic detail of the experiment. A 300 mm focal length fused silica lens focused the laser beam on the target. A beam-scanning arrangement was used to enhance uniformity of ablation and deposition. The distance between target and substrate was set at ~12 mm in all experiments. The laser beam spot area on the target was ~1 mm^2^ and could be tuned by setting the lens focus position. The pulse energy was monitored by an energy-meter Gentec XLP12-3S-H2-D0 Thermopile detector (GENTEC-EO Inc., Quebec, QC, Canada) and the respective fluence (energy density) on the target was controlled in the range of 1 mJ cm^−2^ to 300 mJ cm^−2^ using optical attenuators. All experiments were performed at room-temperature. Figure 1b and c present, respectively, targets used for cellulose and PMMA/YAG:Ce phosphor PLD. SEM images of the polymer composite target shown in Figure 1c, and the as-prepared phosphor powder are depicted, respectively, in Figure 1d,e.

The specific PLD conditions applied for cellulose and polymer/YAG:Ce are discussed in the following subsections and respective parameters are tabulated. We reiterate here that laser wavelength, energy density on target and pulse duration are the most important parameters, as they directly relate to the physical processes involved and the properties of the produced film. First, the photon energy determines the nature of ablation. Infrared wavelengths produce thermal vaporization and pyrolytic effects, while ultraviolet wavelengths yield photodissociation and scissoring of macromolecular chains. Second, the radiation energy density (fluence) on-target and the radiation intensity determine the mass and the energy of the ejected material. Experimentally, for deep ultraviolet ArF excimer laser pulses, a relatively low pulse-energy producing < 20 mJ cm^−2^ fluence on-target is seen to be quite adequate to form durable polymer films at room temperature on practically any kind of closely positioned (~15 mm) substrate.

Parent and grown materials were characterized by optical and scanning electron microscopy (SEM) Zeiss EVO MA10 (Zeiss, Jena, Germany) equipped with Energy Dispersive X-ray (EDX) analysis (Oxford Instruments INCAx-act). Ultraviolet-Visible spectrometry Perkin Elmer Lambda 35 (Perkin Elmer, Waltham, MA, USA), and Shimazu UV-1900 (Shimazu Corporation, Kyoto, Japan) and FTIR spectroscopy Shimazu IRTracer-100 (Shimazu Corporation, Kyoto, Japan) were also used to characterize the polymer structures before and after deposition. Planar films were also formed for reference and analyzed using a Scanning Probe Microscope (Bruker Multi Mode employing the Nanoscope IIIa controller, Santa Barbara, CA, USA). Photoluminescence spectroscopy was performed using a fluorescence spectrophotometer Hitachi F-2500 (Hitachi High Technologies America Inc., Waltham, MA, USA).

Y_3_Al_5_O_12_:Ce is usually excited by InGaN light emitting diodes (LEDs) at 450 nm and emits a broad spectral band from 470 nm to 670 nm and beyond due to the 5d–4f transition of Ce^3+^ ion. To visualize the fluorescence emitted by the polymer/YAG:Ce phosphor coated micro-objects, we used pulsed laser excitation at λ = 450 nm emitted by a Nd:YAG laser source EKSPLA NT340 (EKSPLA, Vilnius, Lithuania) equipped with optical parametric oscillator (OPO). The OPO provided 35 mJ/pulse maximum energy, 4 ns pulse duration, at 450 nm. The incident diverging excitation laser beam had intensity in the range of ~50 MW cm^−2^. The objects were photographed using a 590 nm edge long-wave pass filter.

### 2.2. Cellulose Growth

**Figure 2:**
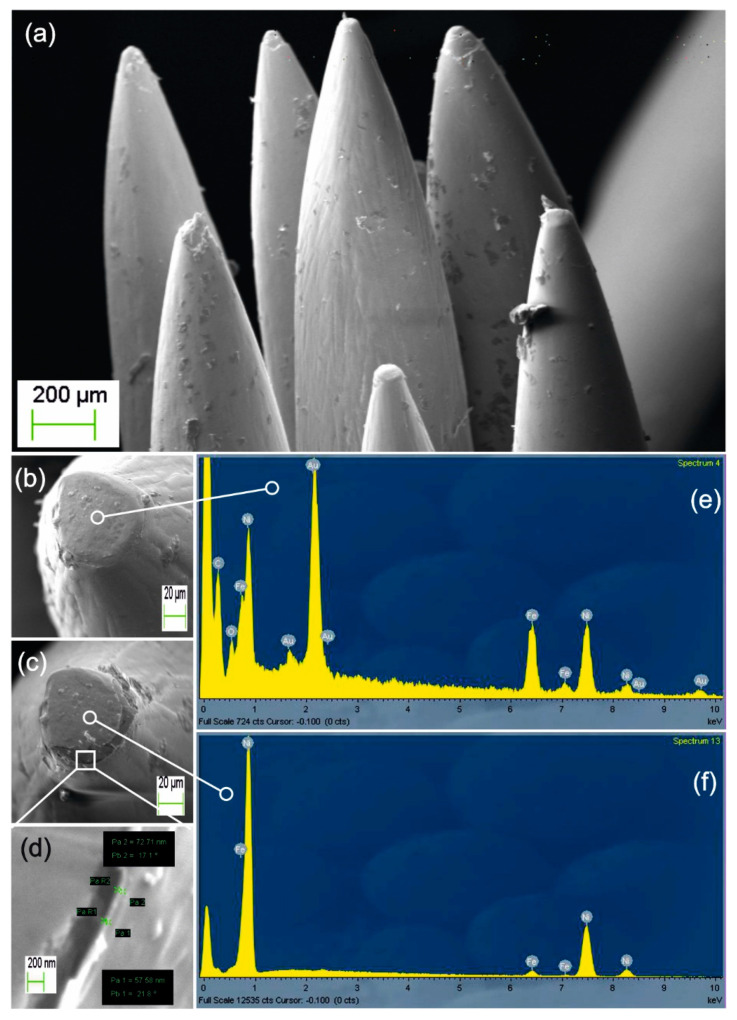
(**a**) Ensemble of cellulose coated stainless-steel needles. (**b**) Detail of a needle tip with as-deposited cellulose layer. (**c**) Image of the same needle with end-tip coating removed mechanically by scalpel blade. (**d**) Close-up view of the cellulose layer edge (frame not in scale) with measured thickness in the range of 50–70 nm. (**e**) EDX analysis of the fully coated tip shown in (**b**) proves the simultaneous presence of hydrocarbon and Fe and Ni steel elements (sputtered Au is used for SEM). (**f**) EDX analysis of uncoated tip shown in (**c**) where hydrocarbon elements are absent.

PLD target pellets made of raw natural cotton harvested in Macedonia, Greece, were used. The harvested crops were processed by careful removal of seeds, manual cleaning and washing in deionized water. The wet fibers were placed in a heating chamber at 80 °C for a period of 24 h for drying. They were subsequently molded in cylindrical pellets of 10 mm diameter and 0.3 mm thickness, by mechanical pressing at 1 GPa and subsequently heated at low pressure (~10 Pa) to remove humidity. This process was repeated several times. No chemical treatment was applied. Figure 1b presents a cotton target used. An ensemble of stainless-steel needles was also used as model 3D micro-object substrate to mimic a hypothetical biomedical instrument (shown in Figure 2a). Applying the results of our previous parametric studies [17], the cotton target was irradiated at the very low fluence of ~8 mJ cm^−2^, corresponding to intensity 1.6 MW cm^−2^, yielding amorphous cellulose films of excellent adherence and optical quality. Table 1 details the conditions applied.

### 2.3. Polymer/Y_3_Al_5_O_12_:Ce Growth

Y_3_Al_5_O_12_:0.06Ce phosphor was prepared by conventional solid-state reaction. Y_2_O_3_ (99.99%), CeO_2_ (99.99%), and Al_2_O_3_ (99.99%) were used as raw materials. All raw materials were thoroughly mixed by milling for 3 h in agate mortars according to the desired stoichiometric ratios. The samples were sintered at 1300 °C for 6 h in alumina crucibles to facilitate the solid-state reaction. The primarily obtained pellets were reground and sintered at 1500 °C to obtain single-phase Y_3_Al_5_O_12_:0.06Ce (Ce:YAG). Using the produced Ce:YAG, two blends of polymer/phosphor composites were prepared and used in this work. Polymethylmethacrylate (PMMA) having M_w_ = 350,000 (Merck, Kenilworth, NJ, USA) and Y_3_Al_5_O_12_:Ce micro-powder were mixed at 10:1 per weight ratio. The homogenized blend was cast in metal mold and placed in an oven at 140 °C for 20 min. A temperature ramp of 1 °C/min down to room temperature followed. In the second case, we mixed PDMS SYLGARD™ 184 (Dow Corning GmbH, Wiesbaden, Germany) with the as-prepared phosphor at 10:1 per-weight ratio, and subsequently cast and cured the blend at 60 °C to solidify. Solid targets of 15 mm diameter and 4 mm thickness were formed in all cases. Figure 1c depicts a new, unused, PMMA/Y_3_Al_5_O_12_:Ce target.

In Figure 1d, the SEM view of its surface depicts the embedded phosphor microparticles, which can be compared with the as-prepared phosphor powder also presented in Figure 1e. The maximum size of particles was ~10 µm. Independent polymer/YAG:Ce synthesis [34] and characterization showed the preservation of photoluminescence properties in polymer matrices [35], as it was also evidenced in our work.

The formed composite targets were ablated by ArF laser pulses at fluence values of up to ~200 mJ cm^−2^ and found to withstand the radiation better than cellulose. In effect, the laser energy density was balanced appropriately to ‘mill’ the phosphor microparticles by laser ablation, without deteriorating the polymers. The substrates used were polytetrafluoroethylene (PTFE) fibers having 100 µm diameter and PMMA polymer optical fibers (POF) of 500 µm diameter, as well as small engineering metal parts, such as springs, screws and nuts. The experimental conditions applied are presented in Table 2.

## 3. Results

The first experimental results concern the coating of an ensemble of stainless-steel needles with cellulose nanolayers as presented in Figure 2a. According to our previous report [17] the deposited layer is pure amorphous cellulose of excellent optical quality and strength. A close-up view of a needle tip covered with cellulose is depicted in Figure 2b. By using a scalpel blade, we removed the coating to reveal the tip apex shown in Figure 2c. Figure 2d is a blown-up view of the edge (frame not in scale) with measured cellulose thickness in the range of 50–70 nm. In Figure 2e, EDX microanalysis of the fully coated tip shown in Figure 2b verifies the simultaneous presence of hydrocarbon with Fe and Ni elements of the steel substrate. The presence of sputtered Au used for the SEM is also noted. In Figure 2f, we present for comparison the EDX analysis of the revealed apex of Figure 2c, where hydrocarbon elements are totally absent.

**Figure 3:**
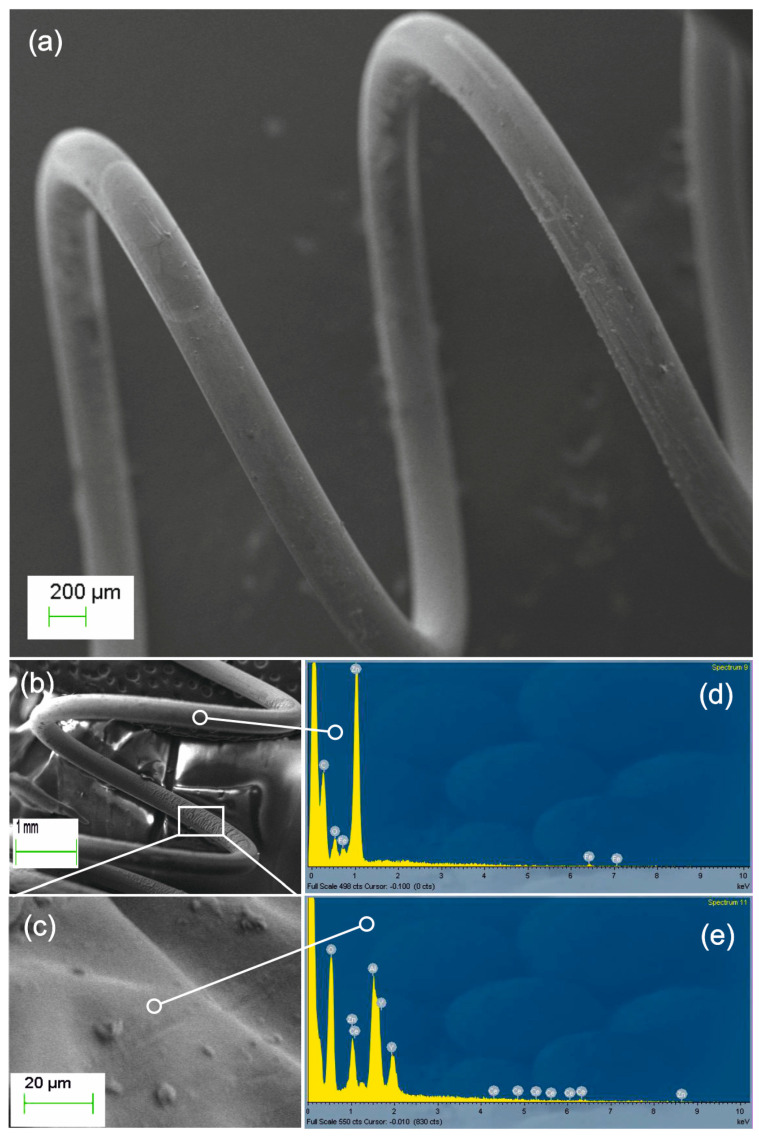
(**a**) SEM image of PMMA/YAG:Ce composite coated steel spring. (**b**) An unevenly coated part of the spring. The thick-coated rippled section (rectangular frame) in the close-up image (**c**) shows delamination. EDX analysis of: (**d**) indicated uncoated section with no phosphor elements, and (**e**) rippled section of (**c**) demonstrating simultaneous presence of the spring (Fe, Zn) and phosphor (Y, Al, Ce) elements.

Our second paradigm demonstrates laser growth of polymer/YAG:Ce nanocomposites on various small 3D metal components. We have used both PMMA and PDMS matrices but focus on the mechanically durable PMMA, even though the elastic properties of PDMS would be advantageous in several applications. Figure 3a presents the SEM imaging of the coated steel spring shown in Figure 1a during the ablation process. By depositing on stationary “spring” substrate, the area pointed in the lower part of Figure 3b was obscured and remained uncoated. In the indicated relatively thick-coated section (in rectangular frame), a rippled delamination fault is found and imaged in the close-up Figure 3c (frame not in scale). Figure 3d presents the EDX analysis of the uncoated section indicated in Figure 3b, verifying the absence of phosphor elements. In Figure 3e, the EDX analysis demonstrates the simultaneous presence of substrate (Fe, Zn) and phosphor (Y, Al, Ce) elements observed in the thick-coated section.

**Figure 4:**
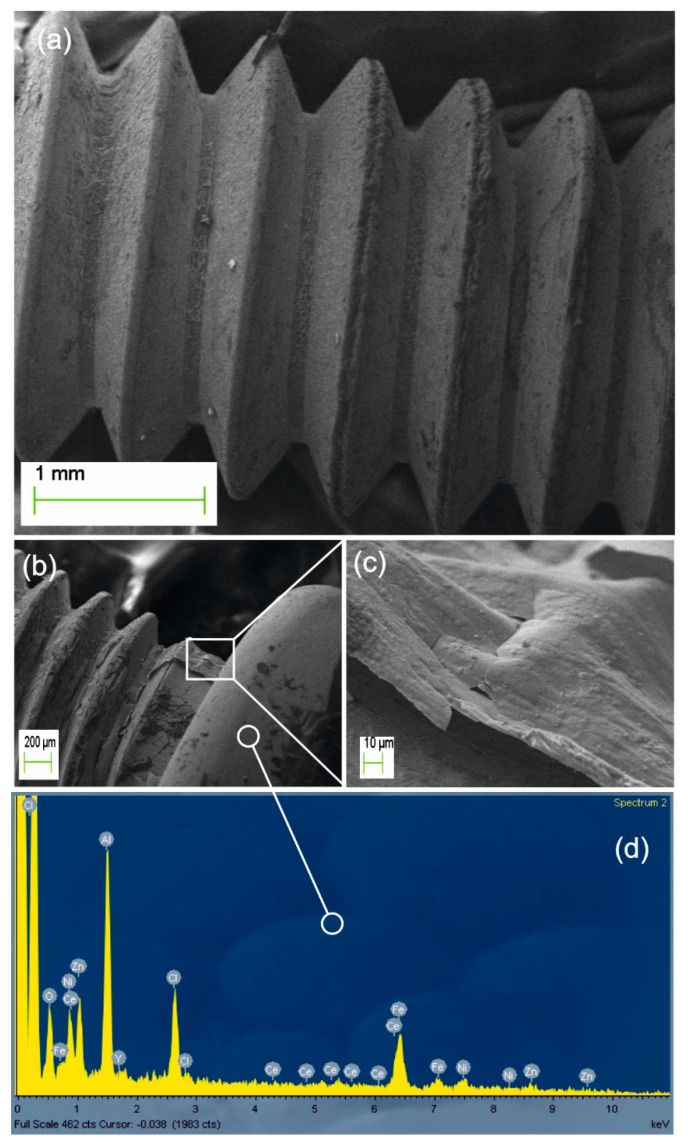
(**a**) SEM image of a socket (Allen) screw coated with PMMA/YAG:Ce. (**b**) Damaged area marked in rectangular frame (not-in-scale) and its close-up image (**c**) showing the exfoliation having ~300 nm thickness. (**d**) EDX analysis of the well-coated screw head confirms the simultaneous presence of phosphor elements (Y, Al, Ce) and the substrate elements (Fe, Ni, Zn) with a strong carbon component (left peaks out of scale).

PMMA/YAG:Ce growth on the more complex stereometry of screw thread is shown in Figure 4a. The accidentally damaged area is framed in Figure 4b. Figure 4c is the close-up view of the latter section (frame not-in-scale) displaying the integrity of the sharp-edged delaminated solid nanolayer having ~300 nm thickness. In Figure 4d, the EDX analysis of the intact coating of the screw-head proves the simultaneous presence of the alloy substrate elements (Fe, Ni, Zn) and the phosphor elements (Y, Al, Ce), together with a strong carbon component (far left peak out of scale).

The above result proves that the Y, Al, and Ce phosphor elements are embedded in the submicron thick polymer matrix. In effect, phosphor clusters and nanoparticles are the expected products of ablation that are transferred, together with the ablated polymer components, to form the nanocomposite film. Naturally, the size of these nanoparticles will be of the order of the layer thickness for a stable film structure to be formed. As we discuss below, the observed fluorescence is a proof of the existence of YAG:Ce nanoparticles forming the functional composite layer. Certainly, this fact does not exclude the presence of isolated elements, molecules or clusters that do not participate in the fluorescence processes.

**Figure 5:**
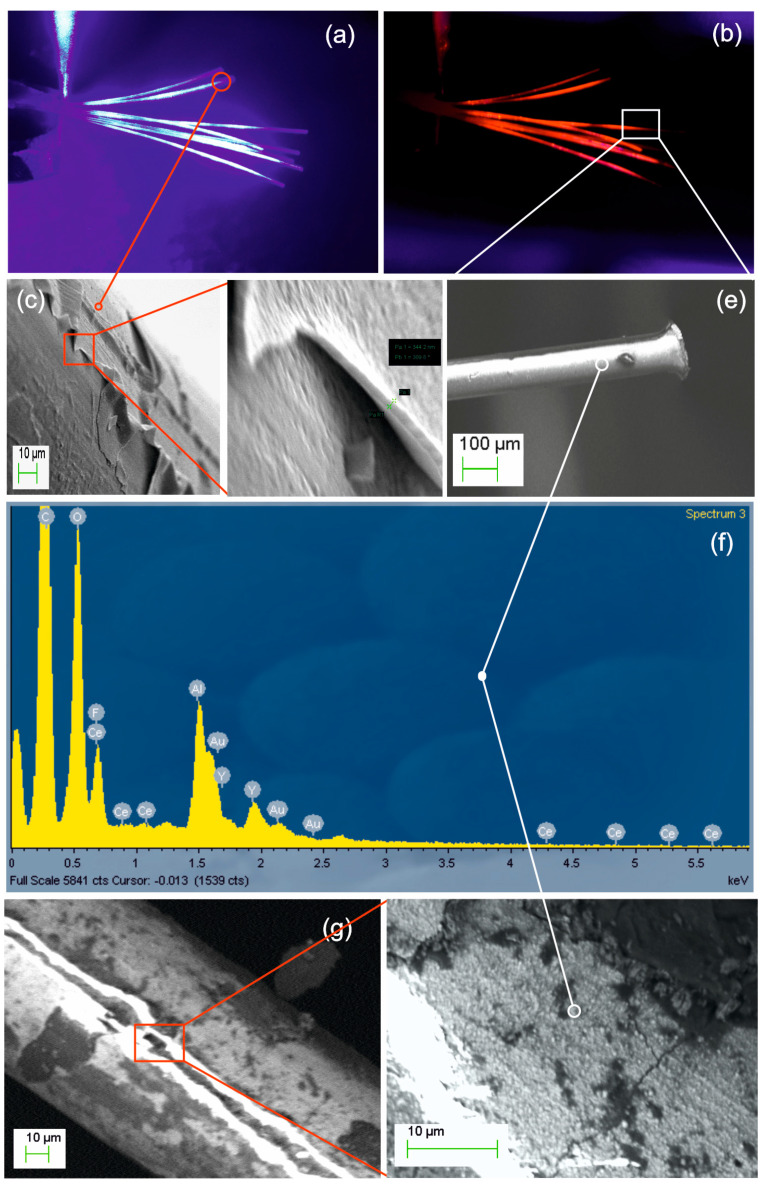
PMMA/YAG:Ce composite deposition on an ensemble of PTFE fibers. (**a**) pulsed laser excitation at λ = 450 nm (**b**). Orange-red fluorescence recorded using a 570 nm long-wave pass filter. (**c**) SEM image of the indicated damaged fiber and (**d**) close-up view of the framed area measuring film thickness at ~350 nm, (**e**) SEM image of a characteristic fiber framed in (**b**) and (**f**) EDX analysis of indicated area showing the simultaneous presence of phosphor elements (Y, Al, Ce) and fluorine and carbon (F, C) components of the PTFE fiber. Backscatter electron (BSE) image of the area (**g**) and close-up view (**h**) depicting intense scattering (white spots) by nanoparticles comprising heavy elements. Rectangular frames are not in scale.

The growth of phosphor/polymer composites on glass and polymer 3D surfaces has also been investigated. Figure 5 presents an example of PMMA/YAG:Ce grown on an ensemble of PTFE fibers of 100 µm diameter. Characterization started with photoluminescence experiments using the fiber ensemble as-coated by PLD. Figure 5a presents a view of the fibers excited by Nd:YAG (OPO) laser pulses at λ = 450 nm. A diverging laser beam was used, and the orange-red fluorescence image was recorded in Figure 5b using the 590 nm edge filter. Following these experiments, the fibers were gold-sputtered and analyzed by SEM and EDX. Figure 5c depicts an SEM image of an exfoliation in the indicated fiber (Figure 5a). The close-up view of the rectangular-framed area is shown in Figure 5d with layer thickness found ~350 nm. In Figure 5e, we focused on the characteristic fiber framed in Figure 5b and performed EDX analysis in the indicated area. The analysis of Figure 5f shows the simultaneous presence of phosphor elements (Y, Al, Ce) and fiber elements fluorine (F) and carbon (C), producing out of range peaks on the left side of scale. The results validate our argument of active phosphor centers, YAG:Ce nanoparticles, embedded in the composite nanolayer. These are produced by ablation of phosphor microparticles blended in the ablation target. In addition, we performed backscatter electron (BSE) SEM imaging in this area, as shown in Figure 5g. A close-up BSE image in the framed section of Figure 5h shows clearly bright nanoparticle scattering centers as expected due to the presence of heavy elements. Further work on the parametric study of nanophosphor formation processes is in progress.

**Figure 6:**
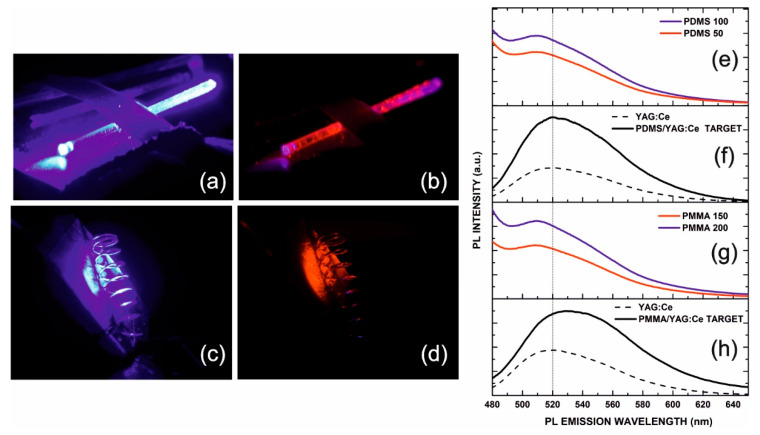
Photoluminescence of two PDMS/phosphor coated objects excited by laser pulses at λ = 450 nm. (**a**) Excitation by radiation is waveguided in the coated POF. (**b**) Respective fluorescence image of PDMS/phosphor coating excited by lossy modes and scattering. The violet coloration observed is a visual (photographic) color mixing effect. (**c**) PMMA/phosphor coated ‘spring’ object illuminated and (**d**) respective fluorescence image of spring and metal holder. Photoluminescence spectra under 450 nm excitation: (**e**) PDMS 50/100 films, respectively, grown at 50 mJ cm^−2^ and 100 mJ cm^−2^, (**f**) composite target (solid line) and as-prepared phosphor (dashed line). (**g**) PMMA 150/200 films, respectively, grown at 150 mJ cm^−2^ and 200 mJ cm^−2^ and (**h**) composite target (solid line) and as-prepared phosphor (dashed line).

Figure 6 demonstrates the observed orange/red fluorescence of coated micro-objects excited by laser pulses at λ = 450 nm. Images are photographed using a 590 nm edge long-wave pass filter. In Figure 6a, a PDMS/phosphor coated plastic optical fiber (POF) is excited by waveguided radiation at 450 nm. Coupling of radiation in the fiber is performed by using a 10× microscope objective in the fiber end positioned in the far right up-corner of the image. The excited lossy guided modes and the respective fluorescence image is recorded in Figure 6b, showing more intense red light scattered from fiber cladding defects. The deep violet coloration observed in this image is a photographic artifact of color mixing at the intense-blue-irradiated areas, such as the fiber ends. In Figure 6c, the metal-spring presented in Figure 1a during the PMMA/phosphor PLD experiment is activated. Figure 6d shows the respective fluorescence image observed. We note that the spring was attached on a metal holder, which was also coated by PLD and fluoresces in the background.

Fluorescence spectra of representative composite PLD targets and nanolayers excited at 450 nm are also presented for comparison. Figure 6e depicts the fluorescence spectrum of the PDMS/YAG:Ce nanolayers grown at laser energy density values 50 mJ cm^−2^ and 100 mJ cm^−2^ (on-target). The respective spectrum of the ablation target used is shown in Figure 6f together with the synthesized phosphor fluorescence (dashed curve). Spectra of the PMMA/phosphor layers grown at laser energy density values 150 mJ cm^−2^ and 200 mJ cm^−2^ (on-target) are also presented in Figure 6g. Respective spectra of the PMMA/phosphor PLD target used and the as-synthesized phosphor are also found in Figure 6h. We note that the fluorescence peak at 520 nm of the raw as-prepared phosphor is maintained in the micro-composite PLD targets with a small ~5 nm red shift observed for PMMA probably due to matrix effects. It is important to underline, however, the observed blue-shift of ~10 nm in the fluorescence emission peak of both PDMS and PMMA nanocomposite films with respect to target and raw phosphor spectra. This effect has been previously observed in the emission of YAG:Ce nanoparticles [35] and it was attributed to the reduced local field of the Ce^+^ ion and the induced decrease in the 4f-state splitting. Similar effects have also been observed in the spectra of YAG:Ce nanoparticles produced by laser ablation in liquid [36]. This latter evidence verifies the presence of active phosphor nanoparticles embedded in the grown matrix and validates the suitability of PLD in the fabrication of hybrid functional nanolayers on 3D surfaces.

## 4. Conclusions

Pulsed laser deposition on 3D object surfaces of complex morphology was demonstrated. Two specific paradigms were presented, respectively addressing the growth of amorphous cellulose and polymer/YAG:Ce phosphor composites using deep-ultraviolet ArF excimer laser radiation (λ = 193 nm). The deposition process takes advantage of the multicomponent ablation producing the congruent transfer of materials from target to substrate. In this work, we used various engineering components and polymer optical fibers as 3D model surfaces that mimic actual photonic and biomedical devices. The formed nanolayers are smooth, well-adhered and conformal to the stereometry of the 3D substrates, even though some faults are visible. Furthermore, they preserve the physical properties of the parent material. In the phosphor nanocomposite paradigm, YAG:Ce nanoparticles produced by ablation are embedded in the co-ablated polymer to form the nanocomposite films. Fluorescence imaging and spectroscopy validated the preservation of functionality in the grown layers. This unique capability of PLD to develop functional nanocomposite structures on surfaces of arbitrary morphology uncovers a great potential for application in the future 3-Dimensional nanotechnologies.

## Figures and Tables

**Table 1:** Specification of experimental conditions applied for Cellulose growth.

Experimental Condition/Parameter	Specification
Target	Natural raw cotton
Substrate	Ensemble of stainless-steel needles
Target—substrate distance	12 mm
Deposition temperature	20 °C
Background pressure	<1 × 10^−4^ Pa
Laser wavelength	193 nm
Laser pulse duration	5 ns
Laser spot size	1 mm^2^
Laser fluence	~8 mJ cm^−2^
Laser pulse repetition rate	100 Hz
Number of pulses nominal per run	360,000

**Table 2:** Specification of experimental conditions applied for PMMA/Y_3_Al_5_O_12_:Ce growth.

Experimental Condition/Parameter	Specification
Targets	PMMA/YAG:Ce and PDMS/YAG:Ce (10:1 w/w)
Substrates	Metal parts/Polymer fibers (dia. 100 µm/500 µm)
Target—substrate distance	12 mm
Deposition temperature	20 °C
Background pressure	<1 × 10^−3^ Pa
Laser wavelength	193 nm
Laser pulse duration	5 ns
Laser spot size	0.9 mm^2^
Laser fluence	PMMA: 50–200 mJ cm^−2^/PDMS: 25–100 mJ cm^−2^
Laser pulse repetition rate	100 Hz
Number of pulses nominal per run	360,000

## Data Availability

Data available on request.
